# Case Report: Testicular Pseudoaneurysm Rupture

**DOI:** 10.5811/cpcem.35400

**Published:** 2025-01-19

**Authors:** Caroline Baber, Eshaan Daas, Michelle Mouri

**Affiliations:** Desert Regional Medical Center, Department of Emergency Medicine, Palm Springs, California

**Keywords:** case report, testicular pseudoaneurysm, GI bleed, prophylactic embolization

## Abstract

**Introduction:**

Gastroduodenal artery embolization is an increasingly common treatment method in patients with upper gastrointestinal (GI) bleeding who fail endoscopy or as a prophylactic procedure to help prevent further episodes. However, this new technique includes new risks including GI tract ischemia and risks associated with endovascular access such as hematoma formation, pseudoaneurysm development, and arterial dissection.

**Case Report:**

We discuss a case of 51-year-old male with recurrent upper GI bleeding who presented to the emergency department for scrotal swelling following the prophylactic embolization of his gastroduodenal artery. He was subsequently found to have a ruptured testicular artery pseudoaneurysm resulting in hemorrhagic shock, which required massive transfusion protocol and vascular repair.

**Conclusion:**

While endovascular access is relatively safe, patients can develop severe complications such as pseudoaneurysm development and subsequent rupture that may not be obviously apparent on physical exam. Because of this, clinicians must have a high index of suspicion for arterial injury, and risk stratification should be used when selecting appropriate candidates for prophylactic procedures.

## INTRODUCTION

Prophylactic embolization of the gastroduodenal artery (GDA) is an increasingly common procedure used in patients with recurrent upper gastrointestinal (GI) bleeding who fail endoscopy.^1^,^2^ This has replaced surgical repair as the next-line treatment.^1^ Long-term success is seen in 50–80% of cases^2^,^3^ with a complication rate of 7–16%.^1^,^2^ The most common complication of this procedure is GI tract ischemia with rare reports of ischemic pancreatitis and multiorgan failure.^1^ Endovascular access can result in additional complications such as hematoma formation, pseudoaneurysm development, and arterial dissection, which occur in up to 8% of cases.^3^ We discuss a case of testicular artery pseudoaneurysm rupture and subsequent hemorrhagic shock as a complication of prophylactic GDA embolization.

## CASE REPORT

**Image 1:**
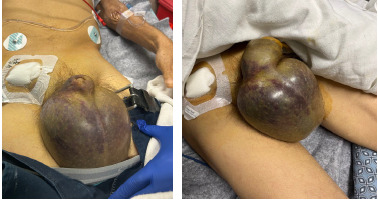
Significant swelling and ecchymosis to the scrotum with recent right femoral artery access site. Left side shows initial presentation, while right side shows progression with extension up the penile shaft two hours later.

A 51-year-old male with a history of hemochromatosis, alcoholic liver cirrhosis, and recurrent upper GI bleeding presented with significant testicular swelling after prophylactic embolization of the GDA via the superior mesenteric artery two days prior. The patient had been home when he heard a “pop” in his abdomen and subsequently developed testicular swelling. His initial vital signs were significant for mild hypotension with a blood pressure of 107/44 millimeters of mercury (mm Hg). Physical exam showed significant swelling and ecchymosis to the scrotum (Image 1). Dorsalis pedis and posterior tibial artery pulses were detected with Doppler ultrasound. The right groin vascular access site was without erythema, induration, or active hemorrhage. During evaluation, the patient became severely hypotensive with a blood pressure of 58/25 mm Hg, and mass transfusion protocol was initiated. Hemostasis was attempted with a 10-pound sandbag and direct pressure over the access site.

**Image 2:**
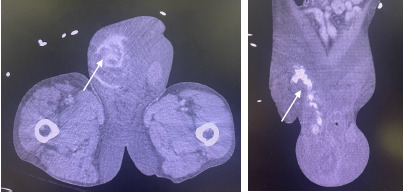
Computed topography angiogram abdomen/pelvis showing 15-centimeter x 3-cm multilocular partially ruptured pseudoaneurysm of the testicular branches of the right external iliac artery involving the right scrotum with adjacent large hematoma and active extravagation of contrast as highlighted by the white arrows.

The patient’s hemoglobin dropped to 5 milligrams per deciliter (mg/dL) from his baseline of 7 mg/dL (reference range 12–15 mg/dL). Computed tomography angiography (CTA) showed a 15-centimeter (cm) x 3 cm multilocular partially ruptured pseudoaneurysm of the testicular branches of the right external iliac artery involving the right scrotum with adjacent large hematoma and active extravasation of contrast (Image 2). Vascular surgery was consulted, and the patient was taken to the operating room for endovascular repair. He was admitted to the intensive care unit and initially was doing well. Five days later, while still hospitalized, the patient was ambulating when he felt a subsequent pop in his right groin and again developed hemorrhagic shock requiring multiple blood transfusions and vasopressor support. A second repair by vascular surgery was planned; however, the patient transitioned to comfort measure and ultimately died two days later.

**CPC-EM Capsule**

What do we already know about this clinical entity?

*Complications of gastroduodenal artery embolization include gastrointestinal ischemia, while complications of vascular access include hematomas and pseudoaneurysms*.

What makes this presentation of disease reportable?

*This was a unique presentation of testicular pseudoaneurysm formation and subsequent rupture resulting in severe hemorrhagic shock*.

What is the major learning point?

*Early recognition of pseudoaneurysm development and prompt vascular repair can prevent mortality and morbidity*.

How might this improve emergency medicine practice?

*This case highlights the potential complications of this procedure and helps physicians consider pseudoaneurysm development/rupture in postoperative patients*.

## DISCUSSION

Embolization of the GDA is generally regarded to be a widely accepted and safe treatment option for patients with recurrent upper GI bleeding or GI bleeding that persists despite endoscopic interventions.^4^ This has widely replaced surgical intervention. Technical success rates of this procedure are 95–100% while long-term success rates, defined as no re-bleeding, occurs 50–80% of the time.^1^ Additionally, patients with significant co-morbidities such as liver cirrhosis or cardiovascular disease may be poor surgical candidates and better suited for a minimally invasive embolization.

The most common complication of this procedure is GI ischemia, which occurs in 7–16% of cases and can progress to multiorgan failure and sepsis.^1^ Other case reports highlight rare complications such as endovascular coil migration into the GI lumen.^4^ However, we must also consider the risks associated with endovascular access. The common femoral artery is the access site most frequently used for various endovascular procedures including arterial embolization and cardiac catheterization.^5^ Complications include hematoma formation, uncontrolled bleeding, pseudoaneurysm development, arteriovenous fistula development, and arterial dissection.^5^

Pseudoaneurysm formation occurs in up to 8% of vascular interventional procedures when the arterial puncture site fails to clot, allowing blood to escape from the vascular lumen.^3^ Commonly, pseudoaneurysms require a high clinical suspicion for diagnosis and may not be apparent if small. The characteristic finding of a pulsatile mass, palpable thrill, and audible murmur is commonly seen,^6^ with the presence of a pulsatile groin mass having the highest positive predictive value for pseudoaneurysm diagnosis.^7^ The risk of pseudoaneurysm development increases significantly when platelet counts are fewer than 200,000 per microliter.^7^ It is essential that pseudoaneurysms be recognized early and treated as they can rupture causing exsanguination and death.^6^

Femoral pseudoaneurysms commonly occur at the bifurcation of the common femoral artery^6^ and rarely within the testicular branches of the external iliac artery as seen this case. Because of the deep location, it is less likely that the common characteristic findings of a pulsatile mass would have been noticeable in our patient. This likely allowed the pseudoaneurysms to grow post-procedurally and symptoms to present only after rupture. Given our patient’s underlying liver disease and coagulopathy, the risk of pseudoaneurysm development was significantly increased. Additionally, the deep location of this artery adds additional challenges with manual compression when compared to pseudoaneurysms at the common femoral artery. Thus, our patient did not present to the emergency department until a large amount of exsanguination had occurred, further increasing the risk of mortality.

## CONCLUSION

While prophylactic embolization of the gastroduodenal artery is relatively safe, it is important for clinicians to be aware of possible life-threatening complications such as pseudoaneurysm development and subsequent rupture. These complications may not be apparent in the immediate post-procedural period and often present vaguely; thus, clinicians must maintain a high index of suspicion for vascular injury following endovascular access. Risk stratification should be used when determining whether a patient is a candidate for such procedures, and emergent vascular surgery intervention should be performed in cases of pseudoaneurysm rupture.
